# The Ribosome Biogenesis Factor Ltv1 Is Essential for Digestive Organ Development and Definitive Hematopoiesis in Zebrafish

**DOI:** 10.3389/fcell.2021.704730

**Published:** 2021-10-07

**Authors:** Chong Zhang, Rui Huang, Xirui Ma, Jiehui Chen, Xinlu Han, Li Li, Lingfei Luo, Hua Ruan, Honghui Huang

**Affiliations:** Key Laboratory of Freshwater Fish Reproduction and Development, Ministry of Education, State Key Laboratory Breeding Base of Eco-Environments and Bio-Resources of the Three Gorges Reservoir Region, School of Life Sciences, Southwest University, Chongqing, China

**Keywords:** Ltv1, ribosome biogenesis, digestive organs, hematopoiesis, P53

## Abstract

Ribosome biogenesis is a fundamental activity in cells. Ribosomal dysfunction underlies a category of diseases called ribosomopathies in humans. The symptomatic characteristics of ribosomopathies often include abnormalities in craniofacial skeletons, digestive organs, and hematopoiesis. Consistently, disruptions of ribosome biogenesis in animals are deleterious to embryonic development with hypoplasia of digestive organs and/or impaired hematopoiesis. In this study, *ltv1*, a gene involved in the small ribosomal subunit assembly, was knocked out in zebrafish by clustered regularly interspaced short palindromic repeats (CRISPRs)/CRISPR associated protein 9 (Cas9) technology. The recessive lethal mutation resulted in disrupted ribosome biogenesis, and *ltv1*^Δ14/Δ14^ embryos displayed hypoplastic craniofacial cartilage, digestive organs, and hematopoiesis. In addition, we showed that the impaired cell proliferation, instead of apoptosis, led to the defects in exocrine pancreas and hematopoietic stem and progenitor cells (HSPCs) in *ltv1*^Δ14/Δ14^ embryos. It was reported that loss of function of genes associated with ribosome biogenesis often caused phenotypes in a P53-dependent manner. In *ltv1*^Δ14/Δ14^ embryos, both P53 protein level and the expression of *p53* target genes, Δ*113p53* and *p21*, were upregulated. However, knockdown of *p53* failed to rescue the phenotypes in *ltv1*^Δ14/Δ14^ larvae. Taken together, our data demonstrate that LTV1 ribosome biogenesis factor (Ltv1) plays an essential role in digestive organs and hematopoiesis development in zebrafish in a P53-independent manner.

## Introduction

The ribosome is a fundamental macromolecular machine, found within all living cells, that synthesizes proteins according to mRNA sequences. Ribosome biogenesis is a very intricate process in cells (Warner, 2001). Eukaryotic ribosome consists of the large 60S and small 40S subunits, which are assembled to form the functional 80S ribosome. In addition to the four ribosomal RNAs (rRNAs) and 82 core ribosomal proteins, which are the components of the 80S ribosome, over 200 non-ribosomal proteins are involved in ribosome biogenesis. This precisely controlled process is inextricably associated with many fundamental cellular activities, such as growth and division (Panse and Johnson, 2010). Disruption of ribosome biogenesis leads to a class of human genetic diseases, collectively termed as ribosomopathies (Narla and Ebert, 2010). Although these diseases are all related to the ribosome dysfunction, ribosomopathies display different clinical manifestations and mechanisms. The symptomatic features of ribosomopathies often include craniofacial defects, digestive organs dysplasia, hematological abnormalities, and the increased risk of some blood cancers (Narla and Ebert, 2010). Ribosomopathies with defects in digestive organs and/or hematological abnormalities include Shwachman-Diamond syndrome (SDS), 5q-syndrome, Diamond-Blackfan anemia (DBA), X-linked dyskeratosis congenita (DC), Treacher Collins syndrome (TCS), and North American Indian childhood cirrhosis (NAIC) (Armistead and Triggs-Raine, 2014).

Numerous genetic models have been established for the investigation of the mechanisms underlying ribosomopathies. In mice, conditional deletion of the syntenic region, including *Rps14*, absent in 5q-syndrome leads to macrocytic anemia, which is the key clinical feature of the disease (Barlow et al., 2010). In zebrafish, knockdown of *rps19* expression causes hematopoietic and developmental abnormalities that is similar to the symptoms of DBA (Danilova et al., 2008). Besides DBA, zebrafish models of SDS (Provost et al., 2012; Carapito et al., 2017; Oyarbide et al., 2020), 5q-syndrome (Ear et al., 2016), NAIC (Wilkins et al., 2013), and DC (Zhang et al., 2012; Anchelin et al., 2013) were generated and characterized. These models are valuable resources to develop potential therapies of ribosomopathies according to the underlying mechanisms. In addition to causative genes in ribosomopathies, some other genes involved in ribosome biogenesis were either mutated or knocked down in zebrafish, such as *bms1l* (Wang et al., 2012, 2016), *kri1l* (Jia et al., 2015), *nol9* (Bielczyk-Maczynska et al., 2015), *nom1* (Qin et al., 2014), and *pwp2h* (Boglev et al., 2013). Depletion of these genes causes disrupted rRNA processing and leads to defects in digestive organs and/or hematological abnormalities, which suggest common roles for ribosome biogenesis factors in organogenesis.

Evidences from a number of animal models of ribosomopathies suggest that P53 is often activated in ribosome dysfunction (Danilova et al., 2008, 2011; Jones et al., 2008; Fumagalli et al., 2009; Barlow et al., 2010; Pereboom et al., 2011; Taylor et al., 2012; Zhang et al., 2012; Boglev et al., 2013; Wilkins et al., 2013; Qin et al., 2014; Bielczyk-Maczynska et al., 2015; Ear et al., 2016). In some cases, inhibition of *p53* is able to rescue the phenotypes (Danilova et al., 2008, 2011; Jones et al., 2008; Barlow et al., 2010; Pereboom et al., 2011; Taylor et al., 2012; Zhang et al., 2012; Bielczyk-Maczynska et al., 2015; Ear et al., 2016), but not in others (Provost et al., 2012; Boglev et al., 2013; Jia et al., 2015). These studies suggest that targeting the P53 pathway could be a therapeutic strategy. However, it should be noted that being a tumor suppression gene, *p53* inhibition may increase risk of cancer development.

LTV1 ribosome biogenesis factor (Ltv1) is a non-ribosomal factor required for the processing of 40S ribosomal subunit (Ameismeier et al., 2018; Collins et al., 2018). Alterations of LTV1 can cause aberrant processing of 18S rRNA in yeast, fruit fly and human cells (Seiser et al., 2006; Tafforeau et al., 2011; Ghalei et al., 2015; Kressler et al., 2015). In Δ*LTV1* yeast cells, the accumulation of 18S rRNA precursors (20S, 21S, and 23S rRNA) is evident, accounted by the decreased pre-rRNA cleavage at sites A0, A1, and A2 (Seiser et al., 2006). Similarly, in fruit fly and human cells, LTV1 deficiency leads to increased level of 21S rRNA, hence a reduced production of the final 18S rRNA and eventually a higher than expected ratio of 28S/18S rRNA (Tafforeau et al., 2011; Kressler et al., 2015). Cell growth is inhibited in *LTV1* loss-of-function yeast strains (Seiser et al., 2006). The fruit fly *LTV1* mutant larvae exhibit development delay and lethality at the second larvae stage (Kressler et al., 2015). These studies suggest the conserved role of *LTV1* in ribosome biogenesis and cell growth from yeast to multicellular animals. However, the function of *LTV1* in vertebrate development remains poorly understood.

Here, we reported that knockout of *ltv1* in zebrafish embryo disrupted ribosome biogenesis. The zebrafish *ltv1*^Δ14/Δ14^ larvae displayed aberrant cartilage structure, defects in digestive organs, characterized by smaller size of liver, intestine and exocrine pancreas, and impaired definitive hematopoiesis. Further characterization of *ltv1*^Δ14/Δ14^ larvae showed that the decreased proliferation gave rise to the dysplastic features of exocrine pancreas and hematopoietic stem and progenitor cells (HSPCs). Although P53 and its target genesΔ*113p53* and *p21* were upregulated, knockdown of *p53* failed to rescue the developmental abnormalities in *ltv1*^Δ14/Δ14^ mutant.

## Results

### Craniofacial Cartilage Was Defective in *ltv1*^Δ14/Δ14^ Zebrafish Mutant Embryo

**FIGURE 1:**
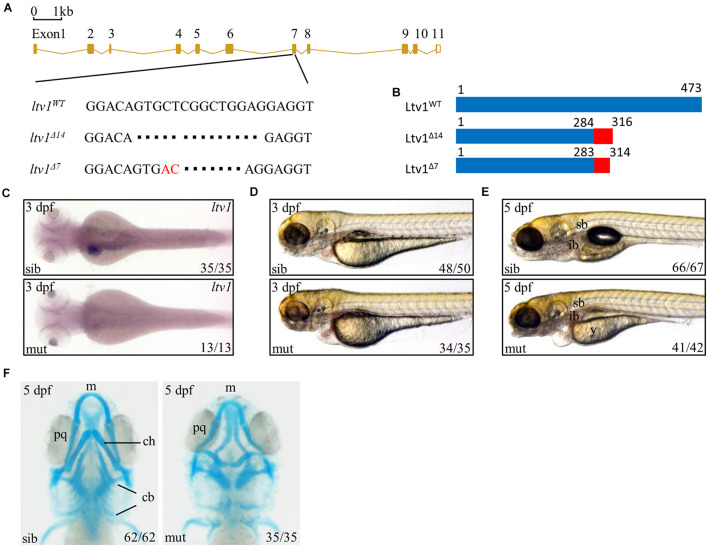
Knockout of *ltv1* and craniofacial defects in *ltv1*^Δ14/Δ14^ mutant. **(A)** The sequences flanking the deletion site in *ltv1* mutants (*ltv1*^Δ7,14^) are shown. The deleted nucleotides are denoted by dashes. In *ltv1*^Δ7/Δ7^ mutant, 9 bp nucleotides are deleted and 2 bp nucleotides (red font) are inserted. **(B)** Two *ltv1* mutant alleles (*ltv1*^Δ14^ and *ltv1*^Δ7^) harbor frameshift mutations that are predicted to give rise to truncated Ltv1 proteins as shown. **(C)** WISH using *ltv1* antisense RNA probe at 3 dpf. **(D,E)** Representative images of *ltv1*^Δ14/Δ14^ mutants and siblings at 3 and 5 dpf. **(F)** Alcian blue-labeled craniofacial cartilage in ventral view at 5 dpf. Abbreviations used: sb, swimming bladder; ib, intestinal bulb; y, yolk; m, Meckel’s; pq, palatoquadrate; ch, ceratohyal; cb, ceratobranchial.

Ltv1 is highly conserved by amino acid sequence homology between human and zebrafish, with approximately 60.9% identity and 76.2% similarity (Supplementary Figure 1). To determine the function of *ltv1*, zebrafish *ltv1*^–/–^ mutants were generated using the clustered regularly interspaced short palindromic repeat (CRISPR)/CRISPR associated protein 9 (Cas9)-mediated approach, and a guide RNA (gRNA) was designed to target the exon 7 of *ltv1*. Two F1 mutant alleles were identified with 14 and 7 bp nucleotides deletion, respectively in the coding region (Figure 1A). Both mutations were predicted to result in frame shifts and premature stop codons in mutant transcripts, encoding two truncated Ltv1 proteins with 284 and 283 N-terminal and 32 and 31 missense amino acids, respectively (Figure 1B). These two mutant alleles could not genetically complement each other, and the *ltv1*^Δ14/Δ14^ mutant allele was used for the following experiments. RNA whole mount *in situ* hybridization (WISH) showed that *ltv1* transcripts were almost absent in *ltv1*^Δ14/Δ14^ mutant at 3 days post fertilization (dpf), indicating that the knockout of *ltv1* was successful (Figure 1C). The mutant mRNA probably underwent a nonsense-mediated decay.

The *ltv1*^Δ14/Δ14^ mutant embryos were morphologically indistinguishable from siblings before 2 dpf with normal blood flow and heart beating. However, at 3 dpf, *ltv1*^Δ14/Δ14^ mutants displayed pericardial edema and aplasia in the head (Figure 1D). At 5 dpf, mutants exhibited underdeveloped intestine, smaller liver, uninflated swim bladder, and impaired yolk absorption (Figure 1E). These phenotypes were completely penetrant and the mutant larvae died from 8 to 11 dpf.

Disruption of ribosome biogenesis can cause abnormal craniofacial skeletons in zebrafish (Mayer and Fishman, 2003; Provost et al., 2012; Qin et al., 2014). Thus, Alcian blue staining was performed to check the craniofacial cartilage structure of *ltv1*^Δ14/Δ14^ mutant. At 5 dpf, mutants displayed severe abnormalities in craniofacial cartilage, including smaller Meckel’s cartilage, curly palatoquadrate and lack of ceratohyal, and five ceratobranchial cartilage (Figure 1F).

### Digestive Organs Were Hypoplastic in *ltv1*^Δ14/Δ14^ Mutant Embryo

**FIGURE 2:**
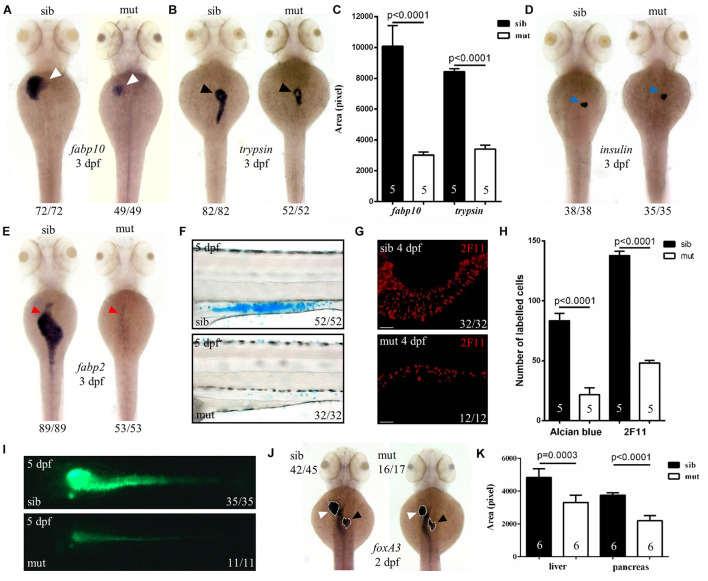
Defects in the development of digestive organs in *ltv1*^Δ14/Δ14^ mutant. **(A,B,D,E)** WISH using the hepatocyte marker *fabp10*, acinar cell marker *trypsin*, beta cell marker *insulin*, and enterocyte marker *fabp2* at 3 dpf. **(C)** Quantification of *fabp10* (siblings, *N* = 5; mutants, *N* = 5) or *trypsin* (siblings, *N* = 5; mutants, *N* = 5) positive area in panel **(A)** or **(B)**, respectively. Bars represent means with SD. **(F)** Alcian blue-stained goblet cells at 5 dpf. **(G)** Enteroendocrine cells (2F11^+^) in the intestine bulb at 4 dpf. **(H)** Quantification of Alcian blue-stained (siblings, *N* = 5; mutants, *N* = 5) or 2F11-positive (siblings, *N* = 5; mutants, *N* = 5) cells in panel **(F)** or **(G)**, respectively. Bars represent means with SD. **(I)** Gut lumen labeled by DCFH-DA at 5 dpf. **(J)** WISH using the pan-endodermal marker *foxA3* at 2 dpf. **(K)** Quantification of the liver and pancreas area labeled by *foxA3* (siblings, *N* = 6; mutants, *N* = 6) in panel **(J)**. Bars represent means with SD. White arrowhead: liver; black arrowhead: exocrine pancreas; blue arrowhead: endocrine pancreas; red arrowhead: intestine.

To further characterize the digestive organ phenotype observed in bright field, WISH was performed to analyze the specific organ formation. Both the liver (marked by *fabp10*) and exocrine pancreas (marked by *trypsin*) of *ltv1*^Δ14/Δ14^ displayed a smaller size compared with sibling at 3 dpf (Figures 2A–C). However, no visible defect was found in the endocrine pancreas (marked by *insulin*) (Figure 2D). In zebrafish, differentiated intestinal cells include three types: enterocytes, goblet cells, and enteroendocrine cells (Chen et al., 2009). In *ltv1*^Δ14/Δ14^ mutant, enterocytes (marked by *fabp2*) at 3 dpf (Figure 2E) and goblet cells (Alcian blue-stained) at 5 dpf (Figures 2F,H) were substantially decreased in number. In zebrafish, both the enteroendocrine and goblet cells of the intestine could be labeled by 2F11 monoclonal antibody (Roach et al., 2013). In zebrafish, goblet cells are only distributed in the posterior part (Roach et al., 2013), not in the intestine bulb, so the 2F11 antibody marked cells in the intestine bulb are enteroendocrine cells. The number of enteroendocrine cells in the intestine bulb was reduced significantly in *ltv1*^Δ14/Δ14^ mutant at 4 dpf (Figures 2G,H). To examine the gut morphology, DCFH-DA, a dye that could label zebrafish gut lumen, was used to visualize the intestine. At 5 dpf, although the overall shape of the mutant intestine resembled that of the sibling, the lumen was narrower than that of the sibling (Figure 2I).

Developmental defects of digestive organs could be due to impaired differentiation of endodermal cells. The genes *foxA1*, *foxA3*, and *gata6* are early endodermal markers that can also label digestive organ primordia in zebrafish (Tao and Peng, 2009). These three genes expressed normally in the *ltv1*^Δ14/Δ14^ mutant endoderm at 1 dpf (data not shown). Both liver and pancreatic buds were found to be smaller in the mutant than that in the sibling, while the intestine seemed normal at 2 dpf (Figures 2J,K and Supplementary Figures 2A,B). These data suggested that the process from the endoderm to bud initiation was intact whereas bud expansion, taking place at a later stage, was affected in the mutant. To test whether liver specification was impaired in the *ltv1*^Δ14/Δ14^ mutant, two of the earliest markers of hepatoblasts, *prox1* and *hhex*, were analyzed (Ober et al., 2006). Consistent with *foxA1*, *foxA3*, and *gata6*, the expression of *prox1* and *hhex* revealed a slightly smaller liver bud in the mutant compared with the sibling at 2 dpf (Supplementary Figures 2C,D). A noticeable hypoplastic liver phenotype in the *ltv1*^Δ14/Δ14^ mutants could be observed at 34 hours post fertilization (hpf) by tracing the *prox1* expression at earlier developmental time points (Supplementary Figures 2E,F). There are two types of glandular tissue in the zebrafish pancreas: exocrine pancreas and endocrine pancreas (Field et al., 2003). By checking *pdx1* (precursor cell of endocrine pancreas), *gcga* (alpha cell), *insulin* (beta cell), and *sst2* (delta cell) expression at 2 dpf, no obvious defect was observed in *ltv1*^Δ14/Δ14^ mutants (Supplementary Figures 2G–J). These data suggested that cell differentiation of endocrine pancreas was not affected in the mutant. However, the number of *ptf1a*^+^ cells (exocrine pancreas progenitor cells) decreased significantly at 2 dpf in the *ltv1*^Δ14/Δ14^ mutant on the *ptf1a:gfp* background (Supplementary Figures 2K,L). Thus, hypoplasia of digestive organs in *ltv1*^Δ14/Δ14^ mutant embryos could be a consequence of impaired organ progenitor cell expansion.

### The *ltv1*^Δ14/Δ14^ Mutation Impaired Definitive Hematopoiesis During Embryogenesis

**FIGURE 3:**
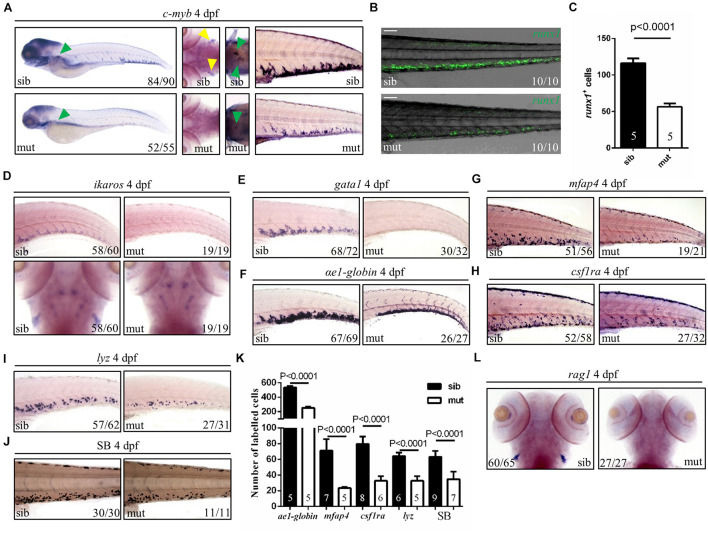
Defects of definitive hematopoiesis in *ltv1*^Δ14/Δ14^ mutant. **(A)** WISH of HSPC marker *c-myb* in the thymus, kidney and CHT at 4 dpf. **(B)** Representative confocal images of *ltv1*^Δ14/Δ14^; *runx1:en-gfp* mutants and siblings at 4 dpf. **(C)** Quantification of *runx1*^+^ cells of *ltv1*^Δ14/Δ14^; *runx1:en-gfp* mutants (*N* = 5) and siblings (*N* = 5) at 4 dpf. Bars represent means with SD. **(D)** WISH of HSPC marker *ikaros* in the CHT and thymus at 4 dpf. **(E–I)** WISH of erythrocyte progenitor marker *gata1* **(E)**, erythrocyte marker α*e1-globin* **(F)**, macrophage markers *mfap4* **(G)** and *csf1ra* **(H)**, and neutrophil marker *lyz* **(I)** in the CHT at 4 dpf. **(J)** Sudan Black-stained neutrophils in the CHT at 4 dpf. **(K)** Quantification of α*e1-globin*, *mfap4*, *csf1ra*, *lyz*, and SB-positive (Sudan Black) cells in panels **(F–J)**, respectively. Numbers of each group are shown in the respective columns. Bars represent means with SD. **(L)** WISH of lymphocyte marker *rag1* at 4 dpf. Yellow arrowhead: thymus; green arrowhead: kidney. Scale bar: 100 μm.

Hematopoietic defects are usually related to the ribosome biogenesis gene deficiency in zebrafish (Oyarbide et al., 2019). Therefore, to figure out the role of *ltv1* in hematopoiesis, different blood cell lineages were examined. HSPCs (marked by *c-myb*, *ikaros*, and *runx1* transgene) in the mutant were pronouncedly reduced in the caudal hematopoietic tissue (CHT), thymus and kidney at 4 dpf (Figures 3A–D). In addition, blood cell lineage markers, such as *gata1* (erythrocyte progenitors), α*e1* (erythrocytes), *mfap4* (macrophages), *csf1ra* (macrophages), *lyz* (neutrophils), *rag1* (lymphocytes), and Sudan Black (neutrophils) staining, were all significantly reduced in the *ltv1*^Δ14/Δ14^ mutant at 4 dpf (Figures 3E–L), indicating the impaired development of definitive erythrocytes, myeloid cells, and lymphocytes.

Two waves of hematopoiesis are involved in zebrafish: the primitive wave and the definitive wave (Jagannathan-Bogdan and Zon, 2013). To assess the status of primitive hematopoiesis in the *ltv1*^Δ14/Δ14^ mutant, two genes regulating the primitive erythroid and myeloid fates, *gata1* and *pu.1*, were examined using WISH at 20 and 22 hpf, respectively, and no visible defect was observed in the mutant (Supplementary Figures 3A,B). The results of *c-myb* expression from 2 to 4 dpf showed that a decreased *c-myb* expression was detectable starting from 3 dpf (Figure 3A and Supplementary Figures 4A–C). Taken together, the primitive hematopoiesis was unaffected while the definitive hematopoiesis was impaired in the *ltv1*^Δ14/Δ14^ mutant, probably due to the reduced HSPCs.

To confirm whether *ltv1* mutation is indeed responsible for the mutant phenotypes observed, zebrafish wild type and mutant form *ltv1* mRNAs were used for rescue experiments. At 3 dpf, both the smaller liver and reduced HSPC phenotypes in the *ltv1*^Δ14/Δ14^ mutant were rescued by zebrafish wild-type *ltv1* mRNA efficiently but not by the mutant form (Supplementary Figures 5A–D).

### Proliferation of Exocrine Pancreas Progenitor Cells and Hematopoietic Stem and Progenitor Cells in *ltv1*^Δ14/Δ14^ Mutant Embryo Was Significantly Reduced

**FIGURE 4:**
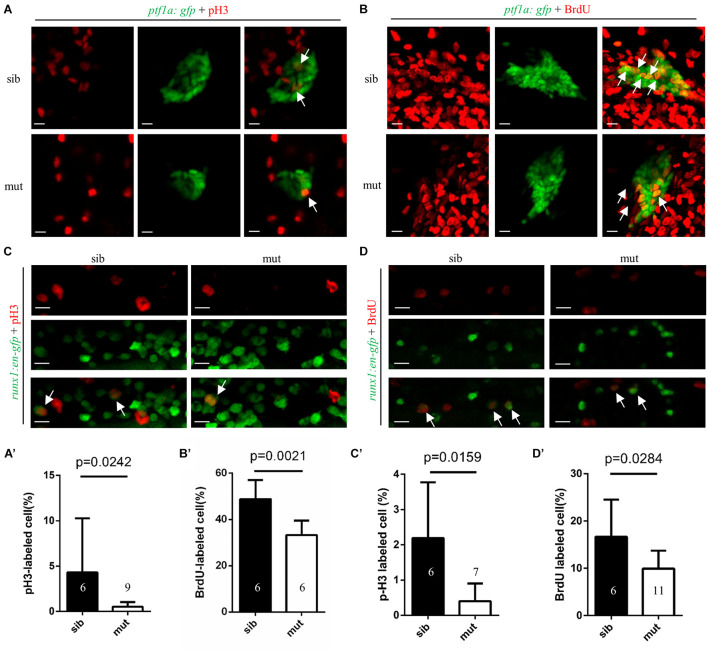
Impaired proliferation of exocrine pancreas progenitors and HSPCs in *ltv1*^Δ14/Δ14^ mutant. **(A,B)** Representative confocal images of pH3 immunostaining **(A)** or BrdU labeling **(B)** in *ltv1*^Δ14/Δ14^; *ptf1a:gfp* mutants and siblings at 2 dpf. **(A’,B’)** The percentage of pH3^+^ **(A’**, siblings, *N* = 6; mutants, *N* = 9) or BrdU^+^ (**B’**, siblings, *N* = 6; mutants, *N* = 6) cells within the *ptf1a*^+^ population in *ltv1*^Δ14/Δ14^ mutants and siblings at 2 dpf. Bars represent means with SD. **(C,D)** Representative confocal images of pH3 immunostaining **(C)** and BrdU labeling **(D)** in *ltv1*^Δ14/Δ14^; *runx1:en-gfp* mutants and siblings at 2.5 dpf. **(C’,D’)** The percentage of pH3^+^ (**C’**, siblings, *N* = 6; mutants, *N* = 7) and BrdU^+^ (**D’**, siblings, *N* = 6; mutants, *N* = 11) cells within the *runx1*^+^ population in *ltv1*^Δ14/Δ14^ mutants and siblings at 2.5 dpf. Bars represent means with SD. White arrow: merged cell. Scale bar: 10 μm.

Disrupted cell proliferation and/or enhanced apoptosis may account for the digestive organs and hematopoiesis defects. The terminal deoxynucleotidyl transferase dUTP nick end labeling (TUNEL) assay revealed no apoptotic cell in the pancreas region in sectioned *ltv1*^Δ14/Δ14^ mutant and sibling at 3 dpf, indicating that apoptosis is not the reason of the dysplastic exocrine pancreas in the mutant (Supplementary Figure 6A). In order to detect the level of cell proliferation, phospho-Histone H3 (pH3) immunostaining and bromodeoxyuridine (BrdU) labeling experiments were performed in mutants and siblings on *ptf1a:gfp* background at 2 dpf. In *ltv1*^Δ14/Δ14^ mutants, both pH3 and BrdU-labeled *ptf1a*^+^ cells were significantly reduced after normalizing for total pancreatic cells (Figures 4A,B,A’,B’). Thus, the impaired exocrine pancreas in *ltv1*^Δ14/Δ14^ mutants would be most likely due to the decreased cell proliferation of the exocrine pancreas progenitor cells.

Consistent with the results observed in the exocrine pancreas, TUNEL assay revealed similar apoptotic level of HSPCs in the CHT between *ltv1*^Δ14/Δ14^ mutants and siblings at 2.5 dpf (Supplementary Figures 6B,C). The proliferation of HSPCs was also reduced in *ltv1*^Δ14/Δ14^ mutants as indicated by the decreased pH3 and BrdU signals of HSPCs in the CHT at 2.5 dpf (Figures 4C,D,C’,D’). Thus, defects in the definitive hematopoiesis were most likely attributed to decreased proliferation of HSPCs, instead of cell death.

### *ltv1* Expression Was Enriched in Digestive Organs During Embryogenesis

**FIGURE 5:**
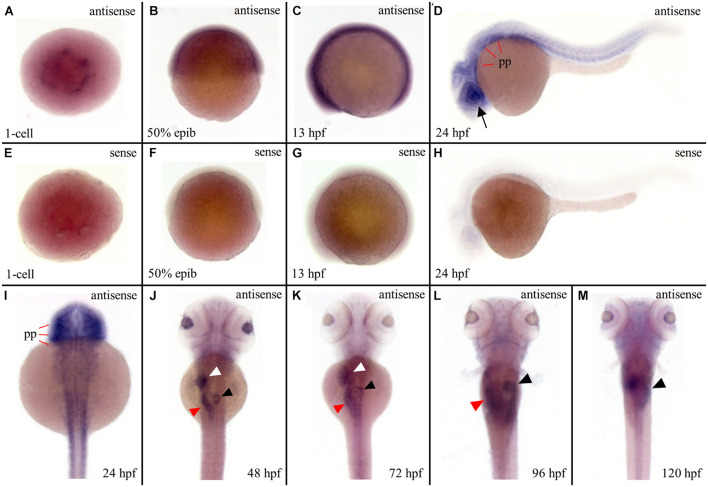
Strong expression of *ltv1* in digestive organs. **(A,I–M)** Dorsal views. **(B–D)** Lateral views, anterior to the left. **(A–D,I–M)** WISH using *ltv1* antisense probe in wild-type embryos at the stages as indicated in each panel. **(E–H)** As a negative control, *ltv1* sense probe is used to perform the WISH. Black arrow: eye. White arrowhead: liver. Red arrowhead: intestine. Black arrowhead: exocrine pancreas. pp: pharyngeal primordia.

To investigate the reason behind tissue specificity of the mutant phenotypes observed, the expression pattern of *ltv1* in zebrafish embryos was examined by WISH using the antisense *ltv1* RNA probe. The sense probe was used as a negative control. At one-cell stage, *ltv1* mRNA was easily detected (Figures 5A,E), which suggested that *ltv1* was a maternal expression gene. From 50%-epiboly to 13 hpf, *ltv1* transcripts were distributed ubiquitously (Figures 5B,C), while no positive staining was detected for sense probe (Figures 5F,G). At 24 hpf, *ltv1* transcripts were found in the eyes and pharyngeal primordia (Figures 5D,H,I). Between 48 and 72 hpf, *ltv1* transcripts were abundant in the eyes, liver, intestine, and pancreas (Figures 5J,K). At 96 and 120 hpf, *ltv1* was highly expressed in the exocrine pancreas (Figures 5L,M). The digestive organ and pharyngeal primordia-specific expression pattern of *ltv1* was consistent with the hypoplastic phenotypes of these tissues during embryogenesis in the mutant. HSPCs and differentiated hematopoietic lineages were also affected severely in *ltv1*^Δ14/Δ14^ mutants; however, no clear *ltv1* mRNA signal was detected in the aorta-gonad-mesonephros (AGM) or CHT by WISH using *ltv1* RNA probe from 24 to 120 hpf.

### Ribosome Biogenesis Was Disrupted in *ltv1*^Δ14/Δ14^ Mutant Embryo

**FIGURE 6:**
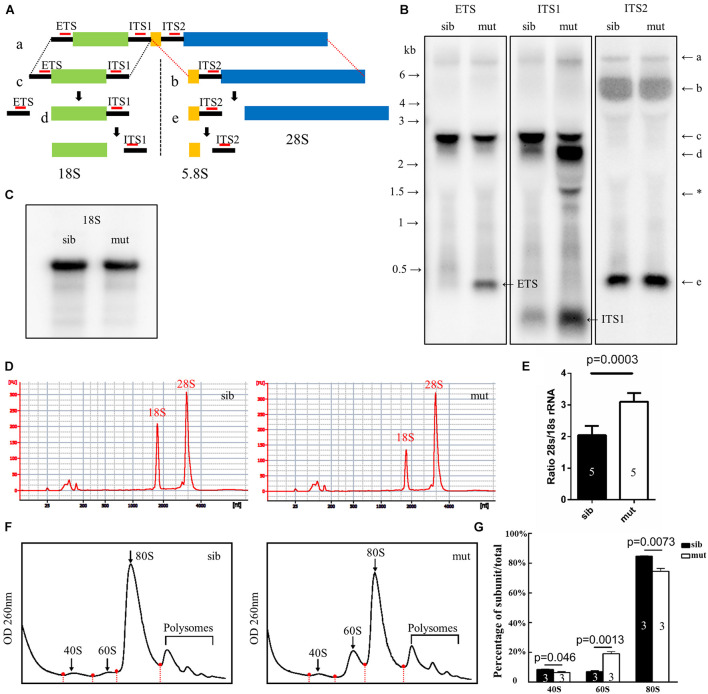
Defects in 18S rRNA processing in *ltv1*^Δ14/Δ14^ mutant. **(A)** Schematic diagram showing the processing pathway of 28S, 18S, and 5.8S rRNAs. The hybridization sites of each probe are indicated by red bars, respectively. **(B,C)** Northern blot analysis using the corresponding probes as indicated to detect 18S rRNA or the intermediate products of rRNA processing. Asterisk: unidentified rRNA intermediate product. **(D)** Representative analysis result by E-bioanalyzer. **(E)** The ratio of 28S/18S rRNA is increased in *ltv1*^Δ14/Δ14^ mutants (*N* = 5), as compared with siblings (*N* = 5). Bars represent means with SD. ETS, external transcribed spacer; ITS, internal transcribed spacer. **(F)** Representative ribosome fractionation results of siblings and *ltv1*^Δ14/Δ14^ mutants at 4 dpf. The peaks of 40S, 60S, and 80S are indicated by arrows. Red dots on the curves represent the lowest points flanking the peaks. The area under respective peaks of 40S, 60S, and 80S, circled by curves, dashed lines, and baselines are measured. **(G)** Percentages of 40S, 60S, and 80S in total lysate in siblings and *ltv1*^Δ14/Δ14^ mutants at 4 dpf. Bars represent means with SD. Quantifications of three independent experiments are analyzed.

In eukaryotic cells, the 28S, 18S, and 5.8S rRNAs are cleaved by various nucleases from a single primary transcript, known as the pre-rRNA. It was reported that deletion of *ltv1* could lead to aberrant processing of 18S rRNA in yeast, fruit fly, and human cells and accumulation of its precursor 20S (yeast) or 21S rRNA (fruit fly and human cells), implying a conserved role of *ltv1* in 18S rRNA processing. To test if it were the case in zebrafish, Northern blot was used to analyze rRNA processing using the probes that could hybridize the ETS, ITS1, ITS2 (ETS/ITS: external/internal-transcribed spacer region), and 18S rRNA (Azuma et al., 2006). ETS, ITS1, and ITS2 probes could mark the rRNA precursor and the intermediate and some minor products (Figure 6A). The ETS and ITS1 probes revealed that the full-length precursor “a” accumulated significantly in *ltv1*^Δ14/Δ14^ mutants, indicating the disruption of rRNA processing (Figure 6B). The “d,” which might correspond to the 20S rRNA in yeast or 21S rRNA in human cells, accumulated while the “c” decreased, showing the impaired 18S rRNA processing in the mutants (Figure 6B). Consistently, the amount of 18S rRNA was declined slightly in the mutants (Figure 6C). Although the “e” increased slightly, the “b” showed no obvious difference in the mutants (Figure 6B), suggesting the intact 28S rRNA processing in *ltv1*^Δ14/Δ14^ mutants. To quantify the amount of 18S and 28S rRNA, E-bioanalyzer analysis was performed and the results showed that the 18S rRNA was reduced obviously in *ltv1*^Δ14/Δ14^ mutants at 5 dpf, while the amount of 28S rRNA remained comparable (Figure 6D). The altered quantity of 18S rRNA therefore caused the imbalance of the 28S/18S ratio in mutants, which is 3.1, compared with 2.0 in siblings (Figure 6E). Consistent with rRNA quantification data, the ribosome fractionation results showed that the amount of 40S subunits and 80S monosomes decreased, while that of the 60S subunits increased about twofold (Figures 6F,G).

### Phenotypes in *ltv1*^Δ14/Δ14^ Mutant Were Independent of P53

**FIGURE 7:**
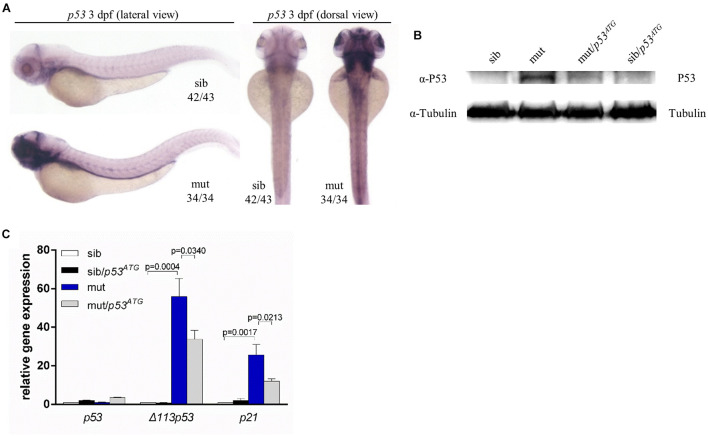
P53 Independence of phenotypes in *ltv1*^Δ14/Δ14^ mutant. **(A)** WISH of *p53* probe for detecting both *p53* and Δ*113p53* at 3 dpf. **(B)** Western blot results of P53 protein of *p53*^*ATG*^ MO-treated or MO-untreated mutants and siblings at 3 dpf. **(C)** Evaluation of expression of *p53* and its transcriptional targets Δ*113p53* and *p21* by quantitative PCR.

A growing number of studies suggest that P53 may play a vital role in phenotypes relevant to ribosome dysfunction (Armistead and Triggs-Raine, 2014). In *ltv1*^Δ14/Δ14^ mutants, there was a clear increase in the expression level of *p53* at 3 dpf, as indicated by WISH using a *p53* probe which can detect both *p53* andΔ*113p53* (Figure 7A). In addition, the P53 protein level was upregulated obviously in mutants (Figure 7B). Then mRNA levels of Δ*113p53* and *p21*, downstream genes of *p53*, were evaluated by quantitative polymerase chain reaction (PCR). Consistently, bothΔ*113p53* and *p21* mRNA levels were increased significantly in *ltv1*^Δ14/Δ14^ mutant, which further suggested the activation of *p53* pathway (Figure 7C). To determine if the downregulation of *p53* could rescue the mutant phenotypes, knockdown of *p53* was achieved by the *p53*^*ATG*^ morpholino injection. The increased P53 expression was attenuated in the mutant, which validated the efficacy of *p53* knockdown (Figure 7B). However, neither the smaller liver nor the reduced HSPC phenotype in *ltv1*^Δ14/Δ14^ mutant could be alleviated by *p53* knockdown (data not shown), suggesting that the mutant phenotypes were independent of P53.

## Discussion

Ltv1 is a non-ribosomal protein essential for 18S rRNA processing in yeast, fruit fly, and human cells (Seiser et al., 2006; Tafforeau et al., 2011; Ghalei et al., 2015; Kressler et al., 2015). In this report, Ltv1 was demonstrated functionally conserved in zebrafish as illustrated by disrupted 18S rRNA processing in the *ltv1* mutants. Deletion of zebrafish *ltv1* resulted in defective growth of liver, exocrine pancreas, intestine, abnormal craniofacial structures and impaired development of HSPCs, definitive erythrocytes, myeloid cells, and lymphocytes. These phenotypic features resembled some specific ribosomopathy models in zebrafish studies (Provost et al., 2012; Carapito et al., 2017; Oyarbide et al., 2019, 2020).

Ltv1 is an assembly factor that can facilitate the incorporation of Rps3 and Rps10 into the small ribosomal subunit in yeast. Ltv1 deficiency led to mispositioned Rps3 in ribosomes (Collins et al., 2018). In zebrafish, knockdown of *rps3* could result in morphological defects, including reduced head size, pericardial edema, and erythropoiesis failure (Yadav et al., 2014). These phenotypic features are consistent with those in *ltv1*^Δ14/Δ14^ zebrafish mutants. Hence, it is interesting to investigate whether *ltv1* functions through *rps3* in digestive system development and hematopoiesis. However, it should be noticed that the morphological defects of *rps3* morphants could be rescued by knockdown of *p53*, while the erythroid failure could not be alleviated (Yadav et al., 2014). In *ltv1*^Δ14/Δ14^ mutants, none of the defects in morphology, digestive organogenesis, or hematopoiesis could be rescued by *p53* knockdown. It was reported that Rps3 could directly interact with P53 and MDM2 (Yadavilli et al., 2009). This may be underlying the P53-dependent recovery of morphological deformities of the *rps3* morphants. Further genetic investigation is required to validate the relationship between *ltv1* and *rps3*. Ribosomes from Ltv1-deficient yeast harbored less Rps10 protein (Collins et al., 2018). Rps10 was found to be mutated in 6.4% of patients with DBA (Doherty et al., 2010). To the edge of our knowledge, no zebrafish mutant of *rps10* has been constructed. It will be meaningful to analyze the phenotypes of *rps10* mutants and investigate the genetic interaction among *ltv1*, *rps3*, and *rps10* in zebrafish.

What is the justification for dysfunction in a macromolecule as ubiquitous and essential as the ribosome causing ribosomopathies with defects in selective tissues? Xue and Barna (2012) believed that the tissue specificity of gene expression in ribosomal biogenesis was the cause. Agreed with this point, *ltv1* was found highly expressed in digestive organs during embryogenesis, which may partially explain the phenotypes of *ltv1* mutants in these organs. However, despite of no detectable expression of *ltv1* in the AGM and CHT, HSPCs and differentiated hematopoietic lineages were impaired severely. Some zebrafish models of ribosomopathy, such as *sbds* (Provost et al., 2012), *rpl11* (Danilova et al., 2011), *rpl24*, *rpl35a* (Yadav et al., 2014), etc., all displayed hematopoietic defects at different levels. While all the respective genes were highly expressed in digestive organs, no description of gene expression in AGM or CHT was reported (Venkatasubramani and Mayer, 2008; Provost et al., 2013), similar to that observed in *ltv1*. One possible explanation is that these genes deficiencies may lead to impaired hematopoiesis indirectly, probably by impairment of the niche of HSPCs. Like *ltv1*, zebrafish *nol9* encoded a non-ribosomal protein, and *nol9* mutants displayed defects in both digestive organs and hematopoiesis. Transmission electron microscopy (TEM) analysis revealed great changes in the CHT niche in *nol9* mutants, including extracellular matrix (ECM) and endothelial cells (Bielczyk-Maczynska et al., 2015).

It has been demonstrated here that Ltv1 is essential for ribosome biogenesis and organogenesis of digestive system and hematopoiesis. Among the existing zebrafish models with deficient ribosome biogenesis, most of them exhibited hypoplasia of liver, pancreas, and intestine, including *nil per os* (*npo*) (Mayer and Fishman, 2003), *titania* (*tti*) (Boglev et al., 2013), *bms1-like* (*bms1l*) (Wang et al., 2012), and *nucleolar protein with MIF4G domain 1* (*nom1*) (Qin et al., 2014), while some displayed defects in definitive hematopoiesis, for example, *kri1l* (Jia et al., 2015). To the best of our knowledge, only one mutant *nol9* (Bielczyk-Maczynska et al., 2015) described both phenotypes. Interestingly, in line with *ltv1*^Δ14/Δ14^ mutants, *nol9* mutants showed arrested development of exocrine pancreas and HSPCs as a result of reduced proliferative rate. Although *nol9* and *ltv1* are involved in the 28S and 18S rRNA processing, respectively, the similar phenotypes in these two models suggest the conserved function of ribosome biogenesis genes during embryogenesis.

Several studies revealed that excess free ribosomal proteins, while ribosome biogenesis was impaired, could outcompete P53 in binding the E3 ubiquitin ligase MDM2, consequently protecting P53 from degradation (Zhang et al., 2003; Dai and Lu, 2004; Dai et al., 2004). In some ribosome biogenesis-deficient models, phenotypes could be rescued by inhibition of P53 (Zhang et al., 2012; Bielczyk-Maczynska et al., 2015; Ear et al., 2016). However, in some other cases, P53-independent cell apoptosis and cell proliferation arrest have also been described (Provost et al., 2012; Boglev et al., 2013; Qin et al., 2014; Yadav et al., 2014; Jia et al., 2015). Although P53 protein and target genesΔ*113p53* and *p21* were upregulated in *ltv1*^Δ14/Δ14^ mutants, knockdown of *p53* could not rescue the defects of the liver or HSPCs, suggesting a *p53*-independent mechanism was involved, which agreed with the fact that the abnormal rRNA processing in LTV1-deficient human cells was P53 independent (Tafforeau et al., 2011). In addition to P53, some other pathways were reported to be involved in the ribosome-deficient zebrafish models. In zebrafish *kri1l* mutants, an increased level of autophagy was observed, and blocking autophagy could significantly restore the definitive hematopoiesis (Jia et al., 2015). In contrast, inhibition of autophagy reduced the lifespan of zebrafish mutants of *pwp2h* gene, which encoded a protein promoting the small ribosomal subunit processing. In *pwp2h* mutants, autophagy was considered a survival mechanism triggered by ribosomal efficiency (Boglev et al., 2013). The question that whether autophagy is involved in the *ltv1* function in zebrafish is required further validation. Rpl35a was mutated in 3.3% DBA (Farrar et al., 2008). In zebrafish *rpl35a* knockdown embryos, upregulation of mammalian target of rapamycin (mTOR) could rescue the morphological defects and the erythroid failure (Yadav et al., 2014). This case suggested that mTOR functioned downstream of *rpl35a*. Urb1, a protein promoting the big ribosomal subunit assembly in zebrafish, was demonstrated to play a role downstream of mTOR in digestive organ formation (He et al., 2017). It is also possible that mTOR pathway is involved in *ltv1*-dependent digestive organ development and hematopoiesis.

In *ltv1*^Δ14/Δ14^ mutants, the proliferation was inhibited and *p53* was activated. It was reported that activation of *p53* could lead to cell cycle arrest *via p21* upregulation (Georgakilas et al., 2017). However, it is possibly not the case in *ltv1*^Δ14/Δ14^ mutants because inhibition of *p53* could not restore the growth of the liver and HSPCs. The P53-independent mechanism underlying the cell cycle arrest might be the key way through which *ltv1* functions in zebrafish. Pescadillo was a protein that played an essential role in 28S rRNA processing and the zebrafish *pescadillo*-deficient embryos displayed underdeveloped liver, gut, and craniofacial cartilage (Allende et al., 1996; Lapik et al., 2004; Provost et al., 2012). Cyclin D1 was indispensable for cell proliferation in cells. As a cyclin-dependent kinase inhibitor, P27 could decrease catalytic activity of cyclin D1 through direct interaction, and so that led to cell cycle arrest (Razavipour et al., 2020). It was reported that the cell cycle arrest in *pescadillo*-deficient cells was due to cyclin D1 downregulation and activation of P27, which was independent of P53 (Li et al., 2009). In erythroid cell lines, ribosome synthesis defects could lead to decreased level of PIM1, a kinase implicated in cell proliferation. The reduction of PIM1 induced cell cycle arrest *via* accumulated P27 in a P53-independent way (Iadevaia et al., 2010). It is interesting to investigate that whether the *ltv1*^Δ14/Δ14^ mutants share the same P27-regulated mechanism, regardless of P53, underlying the cell proliferation inhibition with these two cases.

It should be highlighted that the phenotypes of *ltv1*^Δ14/Δ14^ and *sbds*-deficient embryos share some similar features such as the affected exocrine pancreas and hematopoiesis (Burroughs et al., 2009). Although the Sbds protein plays a role in 60S ribosomal subunit biogenesis (Menne et al., 2007), Ltv1 is required for the assembly of 40S ribosomal subunit (Ameismeier et al., 2018; Collins et al., 2018). The expression pattern of *sbds* is very similar to that of *ltv1* in zebrafish (Venkatasubramani and Mayer, 2008). In line with *ltv1*^Δ14/Δ14^ larvae, morpholino knockdown of *sbds* in zebrafish causes defects in exocrine pancreas, of which could not be rescued by *p53* downregulation, while the endocrine pancreas is normal (Provost et al., 2012). Together with other animal models with ribosome biogenesis dysfunction, *ltv1* zebrafish mutant not only provides new candidate genes for the screening of ribosomopathies with unknown genetic deterioration but also serves as a tool to investigate molecular and cellular mechanisms underlying ribosomal genes deficiency phenotypes. As a powerful model for chemical screen, the corresponding zebrafish mutants may be used for the identification of potential compounds for treating specific ribosomopathies.

## Materials and Methods

### Zebrafish Strains and Embryos Collection

Wild-type Tübingen fish line, transgenic line *ptf1a:gfp* (Godinho et al., 2005), and *runx1:en-gfp* (He et al., 2015) were used and maintained under standard conditions.

### Genomic DNA Extraction

Embryos or fish scales were lysed in the buffer (10 mM Tris–HCl, 50 mM KCl, 0.3% Tween-20, 0.3% NP40, and 1/10 volume proteinase K, Invitrogen, Waltham, MA, United States) at 55°C for 12 h and then the reaction was inactivated by increasing the temperature to 95°C for 20 min. The crude lysate could be used as the template for PCR directly.

### Generation of *ltv1* Mutants by Clustered Regularly Interspaced Short Palindromic Repeat/Cas9 System

The gRNA was designed to target a site in the exon 7 of *ltv1* at the sequence GGACAGTGCTCGGCTGG*AGG* (PAM site in italics). Zebrafish Cas9 mRNA and the *ltv1* gRNA were synthesized as described (Chang et al., 2013; Ear et al., 2016). At one-cell stage, Cas9 mRNA (300 pg) and gRNA (50 pg) were injected into wild-type embryos. At 36 hpf, about 10 embryos were pooled and lysed, and the primers (*ltv1* fw: 5′-TGGTAAGGAGTCTGATTATC-3′ and *ltv1* rv: 5′-CCAATCCATGTGATGCATAC-3′) were used to amplified DNA fragment harboring gRNA targeted site. PCR products were subjected to sequencing to identify potential indels in the region. Upon detecting mutation, the rest of the embryos were raised to adults (F0). Pooled F1 embryos obtained by crossing F0 with wild-type fish were examined for indels in *ltv1* gene using the PCR method described above. The nature of indels could be obtained by sequencing and mutant allele specific primers were then designed according to specific indels.

### Genotyping of *ltv1*^Δ14/Δ14^ Mutants

The common forward primer (*ltv1* fw) was described above. The mutant and wild-type allele-specific reverse primers were listed as follow: wt rv: 5′-CTTTGATGACCTCCTC-3′ and *ltv1*^Δ14^ rv: 5′-CTTTGATGACCTCTGT-3′.

### RNA Whole Amount *in situ* Hybridization

The following digoxigenin-labeled antisense RNA probes were used: *fabp10*, *trypsin*, *insulin*, *fabp2*, *foxA1*, *foxA3*, *gata6*, *hhex*, *prox1*, *pdx1*, *gcga*, *sst2*, *c-myb*, *ikaros*, *gata1*, α*e1-globin*, *mfap4*, *csf1ra*, *lyz*, *rag1*, *apoe*, and *pu.1*. WISH was performed as described previously (Huang et al., 2008; Li et al., 2011).

### Mutant Rescue

Zebrafish wild-type *ltv1* cDNAs were cloned into pCS2 + vector. Mutant form cDNA was obtained by site-directed mutagenesis. The microinjection was performed at one-cell stage, 0.5 ng *in vitro* transcribed either zebrafish wild-type *ltv1* mRNA or *ltv1*^Δ14^ mRNA was used to try to rescue the mutant phenotypes. At 3 days post injection (dpi), embryos were fixed for WISH using the liver-specific *fabp10* or *c-myb* probe.

### Immunochemistry Staining

Immunohistochemistry staining was performed as described previously (Chen et al., 2005). The primary antibodies were goat anti-GFP (Abcam, Cambridge, MA, United States; 1:400), rabbit anti-pH3 (Santa Cruz Biotechnology, Santa Cruz, CA, United States; 1:200), and mouse 2F11 (Abcam, 1:1,000). GFP antibody was used to enhance the GFP signal in *runx1:en-gfp*.

### Bromodeoxyuridine Labeling

For BrdU labeling, BrdU (Roche Diagnostics, Indianapolis, IN, United States; 1 nl, 30 mM) was injected into the pericardium of embryos. Consequently, the embryos were incubated for 1.5–2 h at 28.5°C. After three times of washing with PBST, the embryos were fixed using 4% PFA. After being treated with 2 N HCl for 1 h, the embryos were incubated with mouse anti-BrdU (Roche Diagnostics, United States; 1:50) and goat anti-GFP (Abcam, United States; 1:400) antibodies at 4°C overnight, and finally visualized by Alexa Fluor 555 donkey anti-mouse (Life Technology, Carlsbad, CA, United States; 1:400) and Alexa Fluor 488 donkey anti-goat (Life Technology, United States; 1:400) antibodies.

### TUNEL Assay

Whole embryo and cryosectioned samples were prepared for TUNEL assay, and *in situ* cell death detection kit, TMR Red (Roche Diagnostics, United States) was used following the manuals provided.

### Dyes Staining

Alcian blue, Neutral red, and Sudan black (Sigma-Aldrich, Burlington, MA, United States) staining was conducted as described previously (Herbomel et al., 2001; Le Guyader et al., 2008; Chen et al., 2009; Li et al., 2011). DCFH-DA (Wako, Japan) was used as described previously (Shi et al., 2014).

### Northern Blot

Total RNA was extracted from mutant and sibling embryos at 120 hpf using TriPure Isolation Reagent (Roche Diagnostics, United States). The DIG-labeled DNA probes were PCR-amplified using previously described primers (Azuma et al., 2006). Equal amount of total RNAs were subjected to electrophoresis. The probe hybridization and detection process were carried out as previously described (Chen et al., 2005).

### Quantification of 18S and 28S Ribosomal RNA

Total RNA was extracted from *ltv1*^Δ14/Δ14^ mutants and siblings at 5 dpf. Then, RNA were subjected to E-Bioanalyzer (Agilent 2100) analysis according to the manual.

### Ribosome Fractionation

For each group, 300 embryos at 4 dpf were collected and rinsed by pre-chilled PBS (containing 100 μg/ml cycloheximide) for three times. After removing the yolk through 23 G needles, the embryos were resuspended in 500 μl pre-chilled lysis buffer (5 mM Tris–HCl pH 7.5, 2.5 mM MgCl_2_, 1.5 mM KCl, 100 μg/ml cycloheximide, 2 mM DTT, 0.32 U/μl RNase inhibitor, 0.5% Triton X-100, 0.5% sodium deoxycholate, 1 × EDTA-free Protease Inhibitor Cocktail, Abcam) and sheared on ice through 23, 25, and 27 G needles gradually. Then the lysate was centrifuged at 15,000× *g* for 10 min at 4°C to remove the nuclei and cellular debris pellet. The liquid supernatant was gently loaded on 5–50% gradient sucrose solution (containing 20 mM HEPES pH 7.6, 0.1 M KCl, 5 mM MgCl_2_, 10 μg/ml cycloheximide, 0.1× EDTA-free Protease Inhibitor Cocktail, 32 U/ml RNase inhibitor), which was made by Gradient Master (Biocomp, Gyeongju, South Korea) and centrifuged at 36,000 rpm for 4 h at 4°C in SW41 Ti rotor. The fractions were collected using the Piston Gradient Fractionator (Biocomp) and scanned continuously by Triax Flow Cells detector (Biocomp) to measure the absorbance at 260 nm.

### *p53* Knockdown With Morpholinos

To knock down *p53*, *p53*-morpholino (MO)^*ATG*^ (5′-GCGCCATTGCTTTGCAAGAATTG-3′, Gene Tools, 1 nl, 0.7 mM) was injected to one-cell stage embryos (Chen et al., 2005). The morpholino against human β-*globin* was used as the negative control (Chen et al., 2005).

### Quantitative PCR

Total RNA was extracted from whole embryos using TriPure Isolation Reagent (Roche Diagnostics, United States). cDNAs were generated using Oligo (dT) and SuperScript III reverse transcriptase (Life Technologies, United States). For each sample, three parallel repeated tests were performed. The measured expression of *ef1*α was the internal control for each gene. The primers used: *ef1*α fw: 5′-CTTCTCAGGCTGACTGTGC-3′, *ef1*α rv: 5′-CCGCTAGCATTACCCTCC-3′, *p53* fw: 5′-TGGAGAGGAGGTCGGCAAAATCAA-3′, *p53* rv: 5′-GAC TGCGGGAACCTGAGCCTAAAT-3′, Δ*113p53* fw: 5′-ATAT CCTGGCGAACATTTGGAGGG-3′, Δ*113p53* rv: 5′-CCTCCT GGTCTTGTAATGTCAC-3′, *p21* fw: 5′-GAAGCGCAAA CAGACCAACAT-3′ and *p21* rv: 5′-GCAGCTCAATTACGA TAAAGA-3′.

### Western Blot

*p53*^*ATG*^ MO-injected or *p53*^*ATG*^ MO-uninjected embryos were de-yolked at 3 dpf. Then the embryos were treated with pre-extraction buffer (1 mM EDTA, 0.1 mM Na_3_VO_4_, 20 mM NaF, 1 mM DTT, 100 mM PMSF, and a work concentration of Protease Inhibitor Cocktail in PBS) and lysed in SDS lysis buffer. Subsequently, the lysate was used in Western blot. The primary antibodies used were α-P53 antibody (Abcam, 1:1,000) and α-tubulin antibody (Pierce, 1:500).

### Statistical Methods

The experimental data were analyzed in GraphPad Prism 6.0. The unpaired Student’s *t*-test was used for comparing the means of two groups.

## Data Availability Statement

The original contributions presented in the study are included in the article/Supplementary Material, further inquiries can be directed to the corresponding author/s.

## Ethics Statement

The animal study was reviewed and approved by Institutional Animal Care and Use Committee in Southwest University, China.

## Author Contributions

HH and HR designed the project. CZ, RH, XM, JC, and XH performed the experiments. CZ and HH wrote the manuscript. LLi and LLu commented on the manuscript. All authors contributed to the article and approved the submitted version.

## Conflict of Interest

The authors declare that the research was conducted in the absence of any commercial or financial relationships that could be construed as a potential conflict of interest.

## Publisher’s Note

All claims expressed in this article are solely those of the authors and do not necessarily represent those of their affiliated organizations, or those of the publisher, the editors and the reviewers. Any product that may be evaluated in this article, or claim that may be made by its manufacturer, is not guaranteed or endorsed by the publisher.

## Supplementary Material

Supplementary Figure 1 Ltv1 is conserved among human, mouse and zebrafish. Alignment of Ltv1 protein sequence from human, mouse, and zebrafish.

Supplementary Figure 2 The liver bud growth and exocrine pancreas expansion are affected in *ltv1*^Δ14/Δ14^ mutant. **(A–J)** Embryos at 2 dpf, 34 hpf, and 30 hpf were subjected to WISH to analyze the liver and pancreas formation. The probes used include: pan-endodermal markers *foxA1*, *gata6*; hepatic markers *hhex*, *prox1*; pancreas marker *pdx1*; endocrine pancreas markers *gcga*, *insulin*, and *sst2*. **(K)** Representative confocal images of *ltv1*^Δ14/Δ14^*/ptf1a:gfp* mutants and siblings at 2 dpf. **(L)** Quantification of *ptf1a*^+^ cells of *ltv1*^Δ14/Δ14^*/ptf1a:gfp* mutants and siblings at 2 dpf. Bars represent means with SD. White arrowhead: liver. Blue arrowhead: endocrine pancreas. Scale bar: 10 μm.

Supplementary Figure 3 The primitive hematopoiesis is normal in *ltv1*^Δ14/Δ14^ mutant. **(A,B)** WISH of primitive hematopoiesis regulators *gata1* **(A)** and *pu.1* **(B)** at 20 and 22 hpf, respectively.

Supplementary Figure 4 The defects of HSPC is observable at 3 dpf. **(A)** WISH of *c-myb* in the CHT at 2 dpf. **(B)** Quantification of *c-myb*^+^ HSPCs in the CHT of *ltv1*^Δ14/Δ14^ mutants and siblings at 2 dpf. **(C)** *ltv1*^Δ14/Δ14^ mutants displayed significantly decreased *c-myb* expression in the CHT at 3 dpf compared with the siblings.

Supplementary Figure 5 The mutant phenotypes can be rescued by zebrafish *ltv1* mRNA injection. **(A,C)** Representative images of *fabp10* or *c-myb* expression of unrescued and partially and fully rescued mutants after the injection of zebrafish wild-type *ltv1* mRNA. **(B,D)** The rescue efficiency of the liver or HSPC phenotypes in mutants after the injection of zebrafish wild-type or Δ14 *ltv1* mRNA (*N* ≥ 30 in every group). Bars represent means with SD.

Supplementary Figure 6 Apoptotic level of the exocrine pancreas and HSPCs are not increased in *ltv1*^Δ14/Δ14^ mutant. **(A)** No apoptotic cells are found in the pancreas region in cryosectioned samples from both *ltv1*^Δ14/Δ14^ mutants and siblings at 3 dpf. **(B)** Representative confocal images of the *ltv1*^Δ14/Δ14^*/runx1:en-gfp* mutants and siblings after TUNEL assay at 2.5 dpf. **(C)** Ratio of apoptotic cells in *runx1*^+^ HSPCs in *ltv1*^Δ14/Δ14^ mutants (*N* = 6) and siblings (*N* = 6) at 2.5 dpf. Bars represent means with SD. White arrow: merged cell. Scale bar: 10 μm.
