# Supplementary figures and images for: The Ribosome Biogenesis Factor Ltv1 Is Essential for Digestive Organ Development and Definitive Hematopoiesis in Zebrafish
Source: Front Cell Dev Biol. 2021 Oct 7;9:704730. doi: 10.3389/fcell.2021.704730 (PMC8528963; doi:10.3389/fcell.2021.704730)

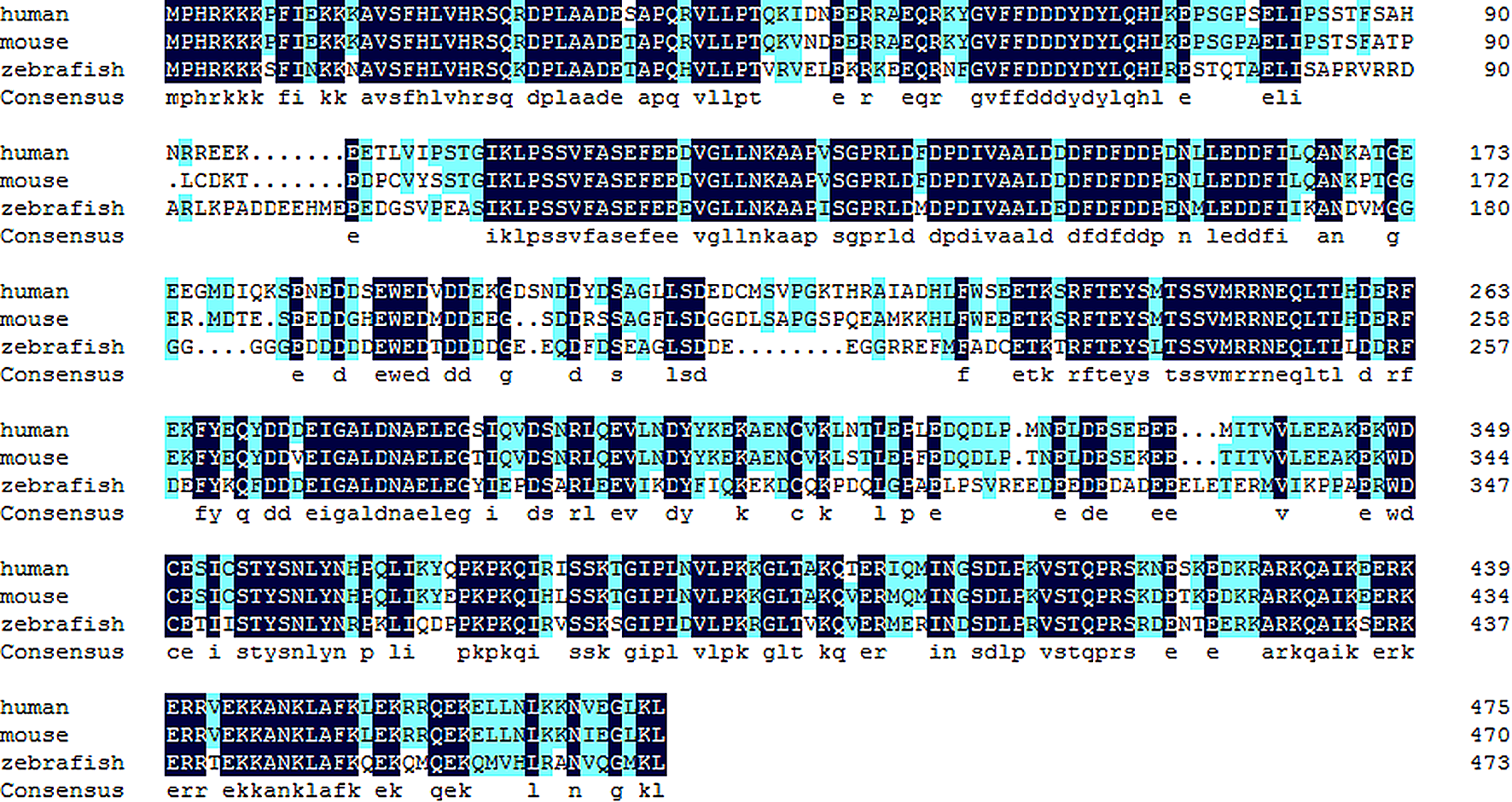

Supplement: Supplementary Figure 1 — Ltv1 is conserved among human, mouse and zebrafish. Alignment of Ltv1 protein sequence from human, mouse, and zebrafish. [file Image_1.TIF]

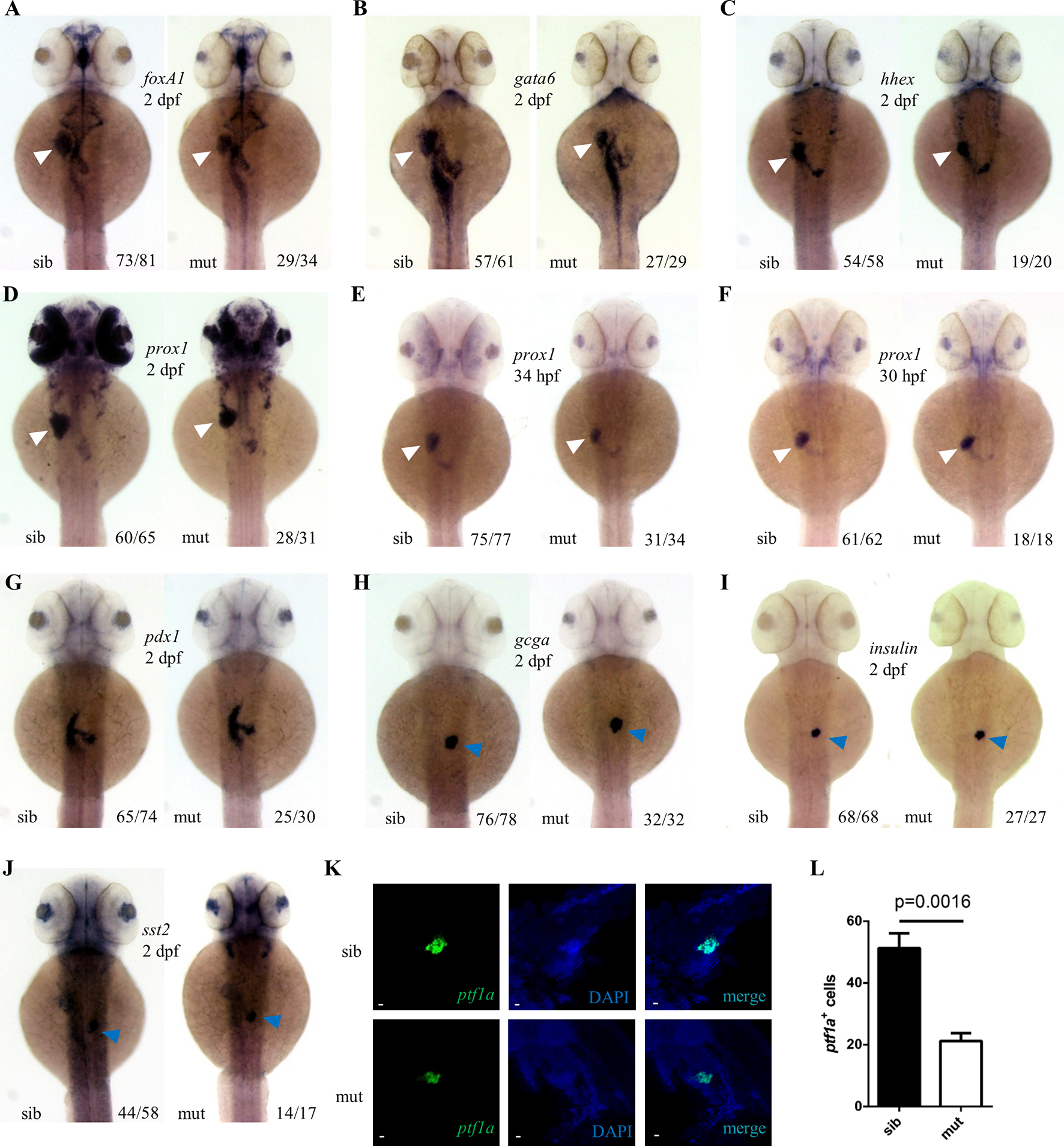

Supplement: Supplementary Figure 2 — The liver bud growth and exocrine pancreas expansion are affected in ltv1Δ14/Δ14 mutant. (A–J) Embryos at 2 dpf, 34 hpf, and 30 hpf were subjected to WISH to analyze the liver and pancreas formation. The probes used include: pan-endodermal markers foxA1, gata6; hepatic markers hhex, prox1; pancreas marker pdx1; endocrine pancreas markers gcga, insulin, and sst2. (K) Representative confocal images of ltv1Δ14/Δ14/ptf1a:gfp mutants and siblings at 2 dpf. (L) Quantification of ptf1a+ cells of ltv1Δ14/Δ14/ptf1a:gfp mutants and siblings at 2 dpf. Bars represent means with SD. White arrowhead: liver. Blue arrowhead: endocrine pancreas. Scale bar: 10 μm. [file Image_2.TIF]

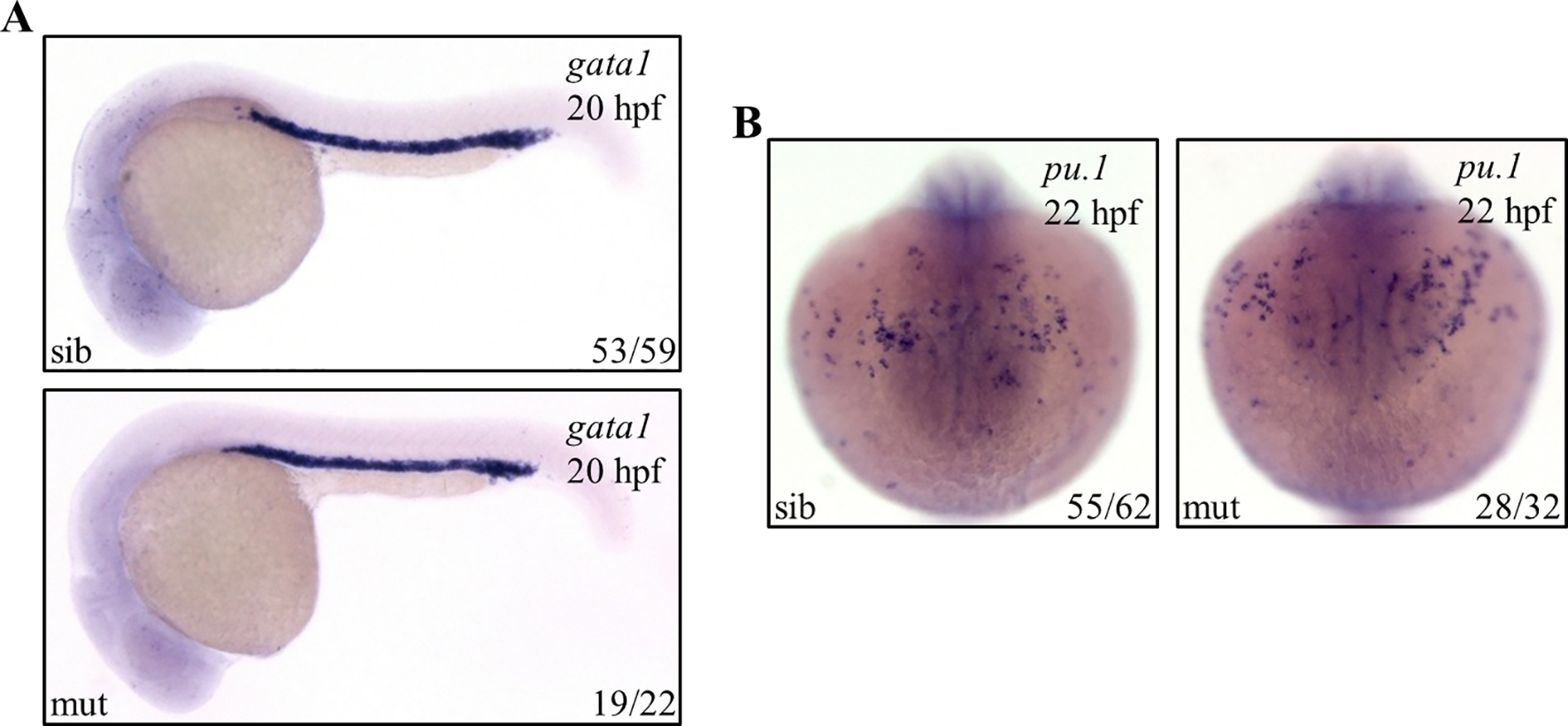

Supplement: Supplementary Figure 3 — The primitive hematopoiesis is normal in ltv1Δ14/Δ14 mutant. (A,B) WISH of primitive hematopoiesis regulators gata1 (A) and pu.1 (B) at 20 and 22 hpf, respectively. [file Image_3.TIF]

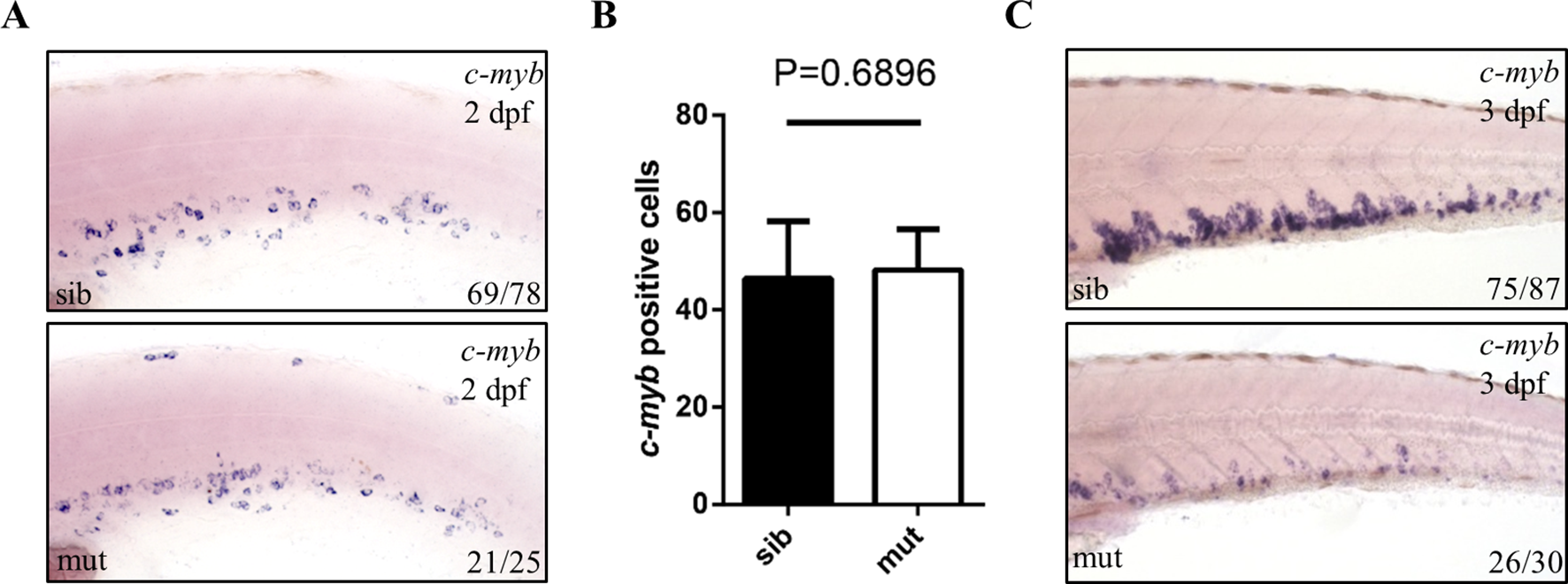

Supplement: Supplementary Figure 4 — The defects of HSPC is observable at 3 dpf. (A) WISH of c-myb in the CHT at 2 dpf. (B) Quantification of c-myb+ HSPCs in the CHT of ltv1Δ14/Δ14 mutants and siblings at 2 dpf. (C) ltv1Δ14/Δ14 mutants displayed significantly decreased c-myb expression in the CHT at 3 dpf compared with the siblings. [file Image_4.TIF]

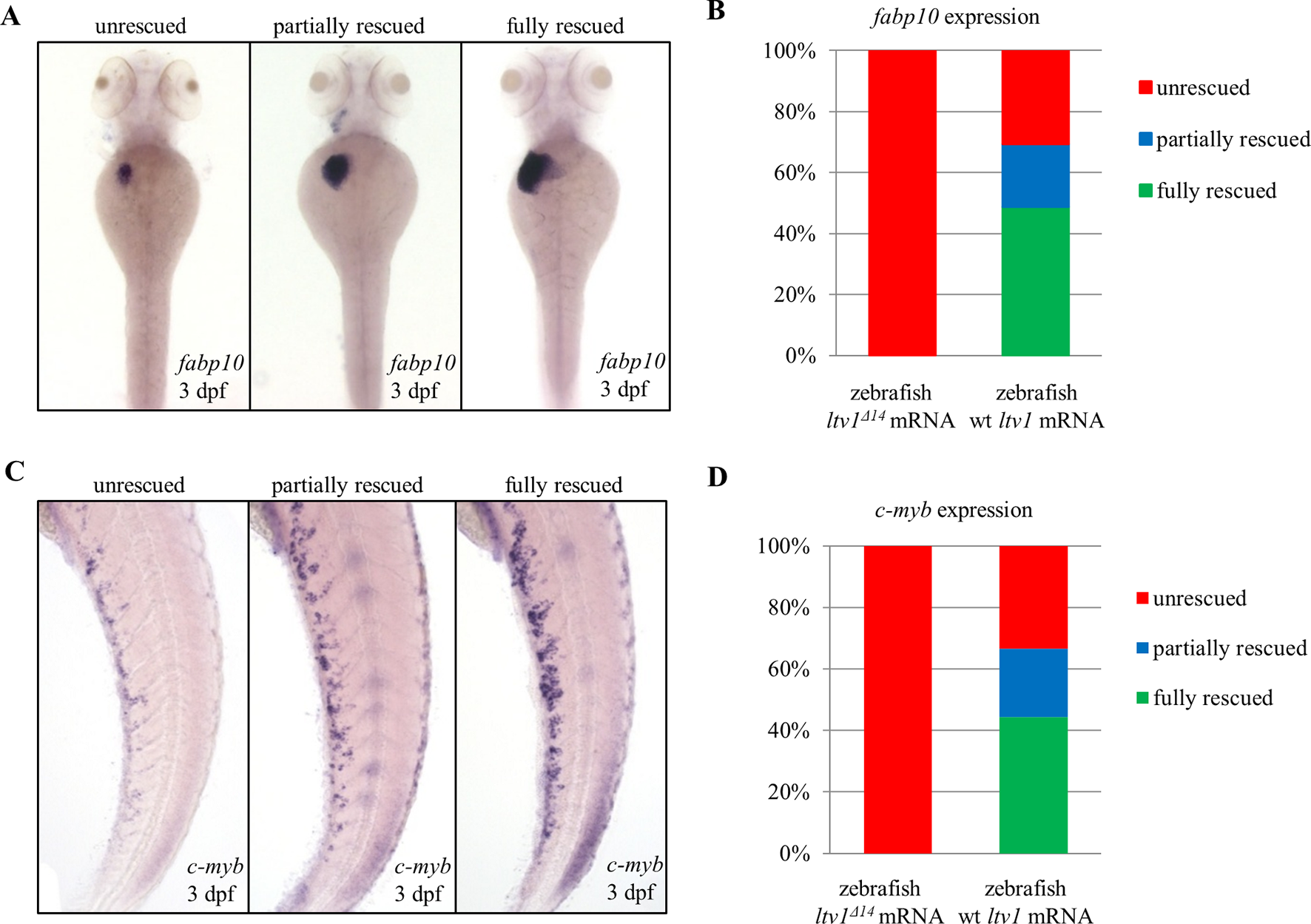

Supplement: Supplementary Figure 5 — The mutant phenotypes can be rescued by zebrafish ltv1 mRNA injection. (A,C) Representative images of fabp10 or c-myb expression of unrescued and partially and fully rescued mutants after the injection of zebrafish wild-type ltv1 mRNA. (B,D) The rescue efficiency of the liver or HSPC phenotypes in mutants after the injection of zebrafish wild-type or Δ14 ltv1 mRNA (N ≥ 30 in every group). Bars represent means with SD. [file Image_5.TIF]

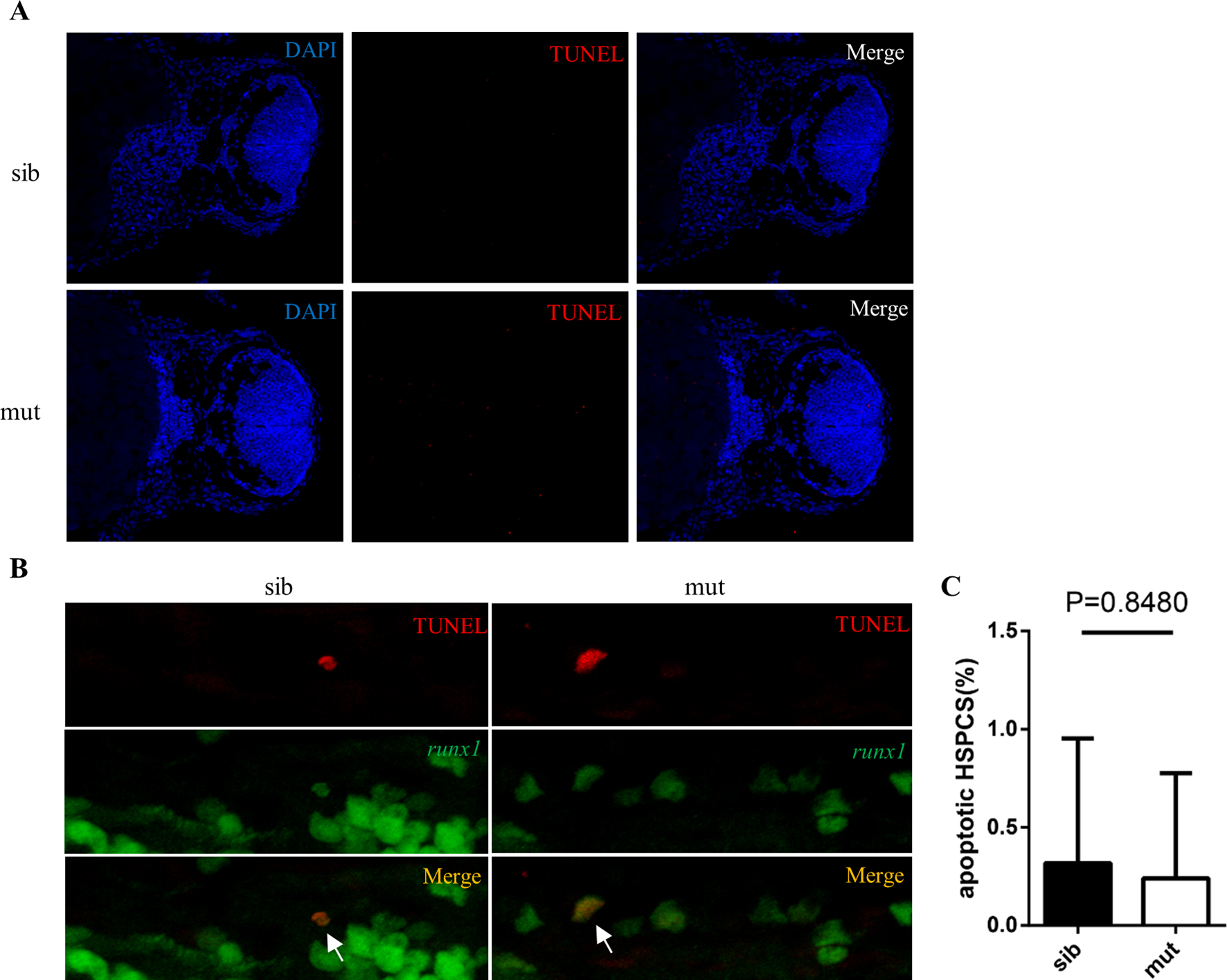

Supplement: Supplementary Figure 6 — Apoptotic level of the exocrine pancreas and HSPCs are not increased in ltv1Δ14/Δ14 mutant. (A) No apoptotic cells are found in the pancreas region in cryosectioned samples from both ltv1Δ14/Δ14 mutants and siblings at 3 dpf. (B) Representative confocal images of the ltv1Δ14/Δ14/runx1:en-gfp mutants and siblings after TUNEL assay at 2.5 dpf. (C) Ratio of apoptotic cells in runx1+ HSPCs in ltv1Δ14/Δ14 mutants (N = 6) and siblings (N = 6) at 2.5 dpf. Bars represent means with SD. White arrow: merged cell. Scale bar: 10 μm. [file Image_6.TIF]
